# X-ray microscopy reveals endophallic structures in a new species of the ground beetle genus *Trechus* Clairville, 1806 from Baltic amber (Coleoptera, Carabidae, Trechini)

**DOI:** 10.3897/zookeys.614.9283

**Published:** 2016-09-01

**Authors:** Joachim Schmidt, Igor Belousov, Peter Michalik

**Affiliations:** 1 University of Rostock, Institute of Biosciences, General and Systematic Zoology, Universitätsplatz 2, 18055 Rostock, Germany; 2 Lindenstraße 3a, 18211 Admannshagen, Germany; 3 All-Russian Institute of Plant Protection, 3, Podbelsky shosse, 196608 St. Petersburg, Russia; 4 Zoological Institute and Museum, Ernst-Moritz-Arndt-University, Loitzer Str. 26, 17489 Greifswald, Germany

**Keywords:** Eocene, micro-CT, 3-D reconstruction

## Abstract

The third fossil species of the genus *Trechus* Clairville, 1806 is described from Baltic amber: *Trechus exhibitorius* **sp. n.** Details of external and internal morphology were analysed using X-ray micro-computed tomography (micro-CT) and important diagnostic features of the internal male genital sac (endophallus) are described in detail for the first time in a fossil ground beetle. Based on these data, we could assign *Trechus exhibitorius* **sp. n.** to *Trechus* sensu stricto and this new fossil species seems to represent a basal branch of a lineage comprising species diverse groups of extant *Trechus* mainly distributed in the Caucasus and Anatolia. Thus, our results support previous studies suggesting that *Trechus* is a phylogenetically old lineage already present in the Eocene with numerous species.

## Introduction

Although the occurrence of ground beetles of the genus *Trechus* Clairville, 1806 in the Eocene Baltic amber was reported already a century ago (Klebs 1910) the first two species were not described at the species level until recently: *Trechus balticus* Schmidt & Faille, 2015, and *Trechus eoanophthalmus* Schmidt, Hoffmann & Michalik, 2016. Both species were identified as representatives of *Trechus* sensu auctorum which, based on molecular data, is a non-monophyletic assemblage (see Faille et al. 2010, 2011, 2013). Due to the absence of male genital characters (the type of *Trechus balticus* is a female; the type of *Trechus eoanophthalmus* is a male but insufficiently preserved) it was argued that the taxonomic position of both fossil species within *Trechus* sensu lato cannot be assessed with certainty (Schmidt and Faille 2015, Schmidt et al. 2016). However, their striking similarity to extant species of the *Trechus* sensu stricto led the authors to conclude that the fossils are likely to be representatives of the stem lineage of the latter group. If this holds true, the origin of *Trechus* sensu stricto has to be assumed in the Paleogene and thus at least 10–20 Mio years earlier than suggested based on molecular analyses (Faille 2013). Thus, the investigation of fossil *Trechus* material with preserved important character, e.g. male genital characters, would hence be of high value for dating the Trechini phylogeny.

In this paper we describe the third wingless Trechini species from Baltic amber. In contrast to the abovementioned fossils, this amber inclusion represents the first ground beetle fossil with well-preserved male genitalia. We investigated this specimen using light microscopy and X-ray micro-computed tomography (micro-CT) for revealing internal genital characters. We discuss the implications of the observed genital characters especially with regard to its placement within one of the extant *Trechus* lineages.

## Material and methods

The specimen was studied and imaged using light microscopy and micro-CT. The methods and technology used were described in detail in a previous work by Schmidt et al. (2016). Micro-CT scans were performed under phase contrast (40 KV, 8 W) using a 4× detector (10 s, 4.15 µm pixel size) and 10× detector (30 s, 1.89 µm pixel size). Additionally, a movie of the volume rendering of the endophallus was obtained using the MovieMaker module in Amira 5.6. The image stacks of the micro-CT scans have been deposited in MorphDBase (www.morphdbase.de/?P_Michalik_20160818-M-8.1; www.morphdbase.de/?P_Michalik_20160818-M-9.1; www.morphdbase.de/?; www.morphdbase.de/?P_Michalik_20160818-M-10.1).

Measurements of the fossil specimen were taken as follows: body size was quantified by the standardized body length, i.e., the sum of: (1) the distance from the apex of the right mandible in closed position to the cervical collar, (2) the median length of the pronotum, (3) the distance from the base of the scutellum along the suture to the apex of the left elytron. The width of the head, of the pronotum, and of the elytra was measured at their widest points. The width of the pronotal apex was measured between the tips of the apical angles, the width of the pronotal base was measured between the tips of the laterobasal angles.

## Taxonomy

### Trechus exhibitorius sp. n.

Taxon classificationAnimaliaColeopteraCarabidae

http://zoobank.org/EE059FA2-2249-4132-A112-4C6A48F3FFAD

Figs 1–3 , 4–6 , 7–12 , 13–14 , 15 , 16

#### Holotype.

**Figures 1–3.**
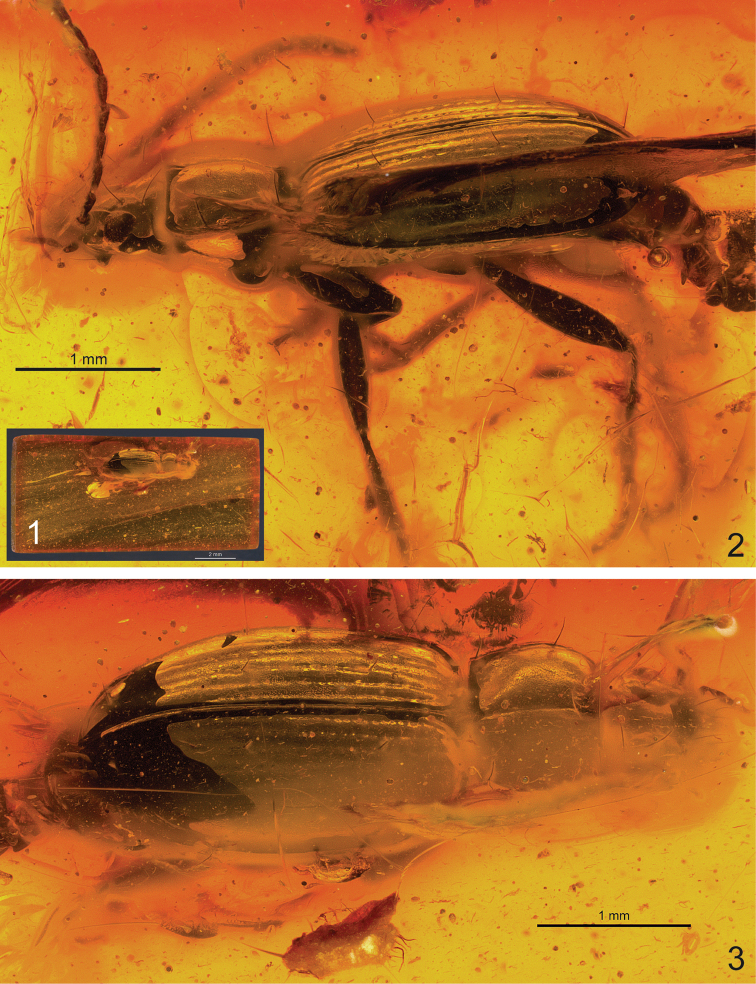
*Trechus exhibitorius* sp. n., light microscopic images of the holotype. **1** general view of the fossil with contours of the amber piece **2** left lateral aspect **3** dorsal aspect.

Male in Baltic amber; size of piece approximately 12 × 5 × 5 mm (Fig. 1), with collection label data “GZG.BST.16192 / (alte Nr. G. 645) / Coleoptera: Carabidae / Bembidium / Geologisch-Paläontologisches Institut und Museum / Göttingen” (front side) and “Species „b“ / Nr.2” (back side), in Geoscience Museum, University of Göttingen, Germany (GMG). This amber piece is part of the former Königsberg Collection which is currently preserved at the GMG. This collection includes pieces of Baltic amber collected in the area of the Curonian Spit up until the first half of the 20^th^ century.

#### Preservation status.

**Figures 4–6.**
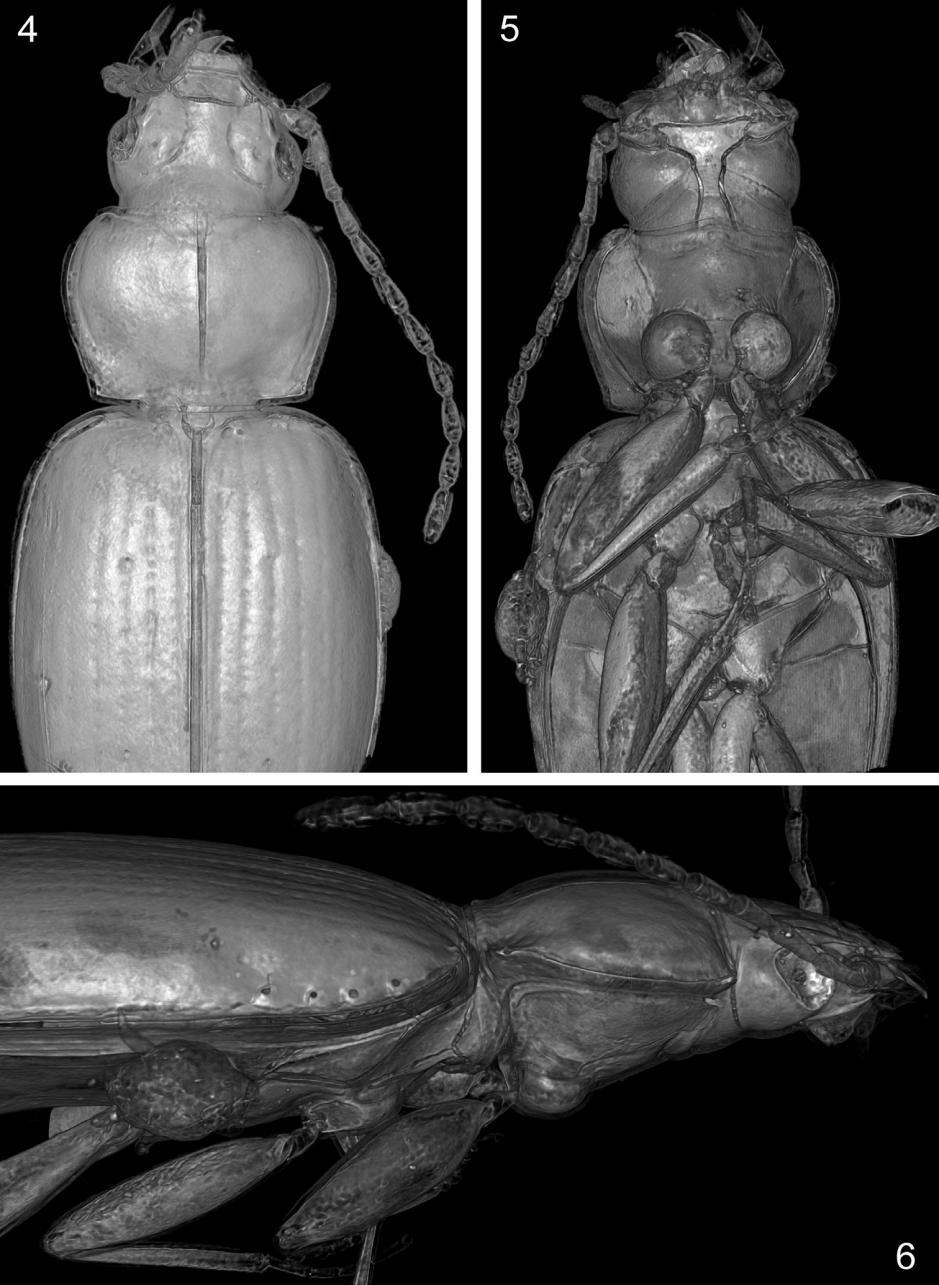
*Trechus exhibitorius* sp. n., volume rendering of the holotype, anterior portion. **4** dorsal aspect **5** ventral aspect **6** right lateral aspect.

**Figures 7–12.**
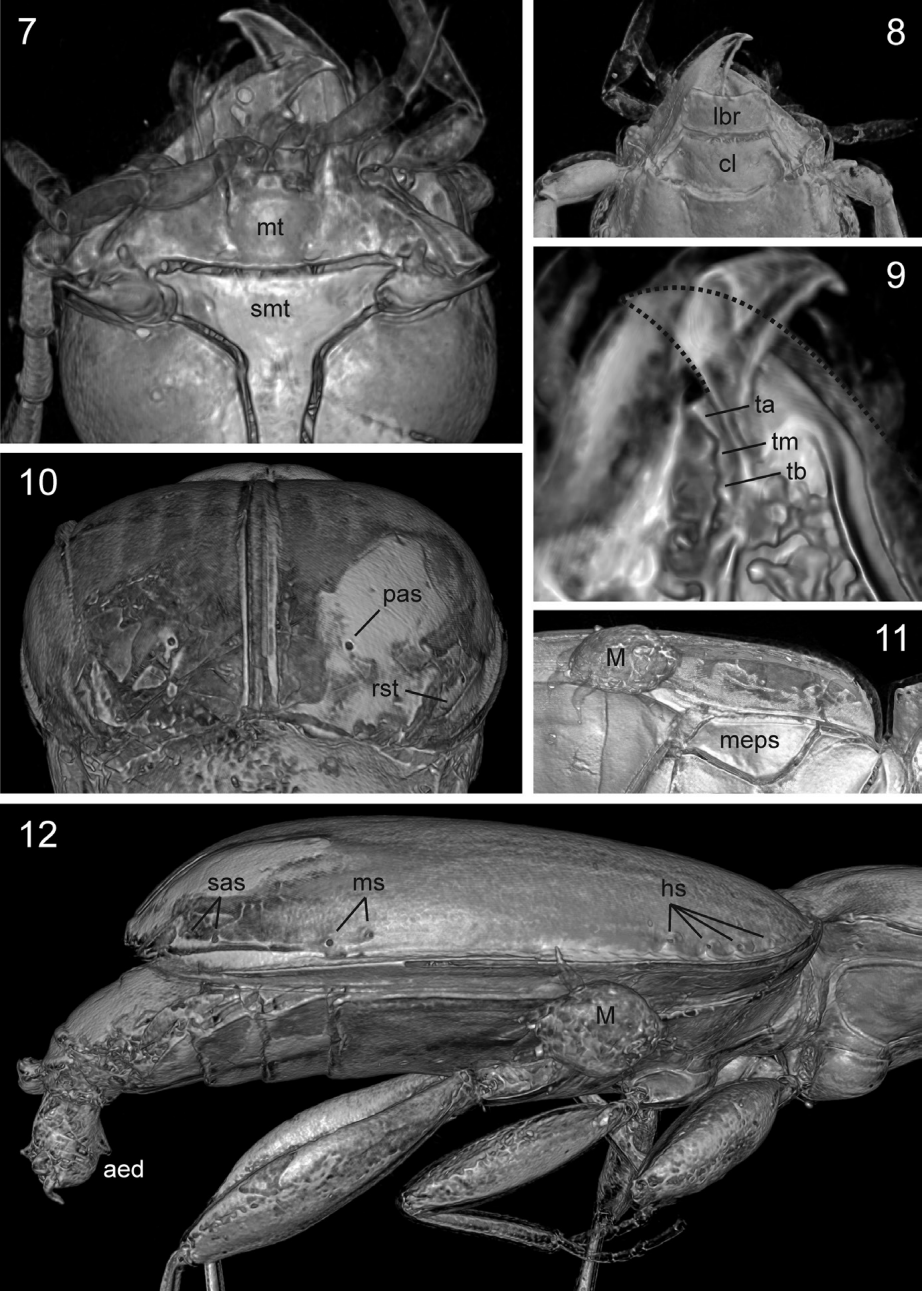
*Trechus exhibitorius* sp. n., holotype, volume rendering of selected body parts. **7** head, ventral aspect **8** head, anterior part, dorsal aspect **9** mandibles, view from dorsal (the dorsal surface of the right mandible is cut together with the labrum in order to show the mandibular dentition; the contour of the apical portion of the right mandible is indicated by a dotted line) **10** elytra, caudal aspect **11** elytral epipleuron and left external part of metathorax **12** elytra and abdomen, right lateral aspect. Abbreviations: aed = aedeagus; cl = clypeus; hs = humeral series; lbr = labrum; M = phoretic mite; meps = metepisternum; ms = medial series; mt = mentum; pas = preapical seta; rst = recurrent stria; sas = subapical series; smt = submentum; ta, tb, tm = apical, basal, medial denticle of the right mandible.

**Figures 13–14.**
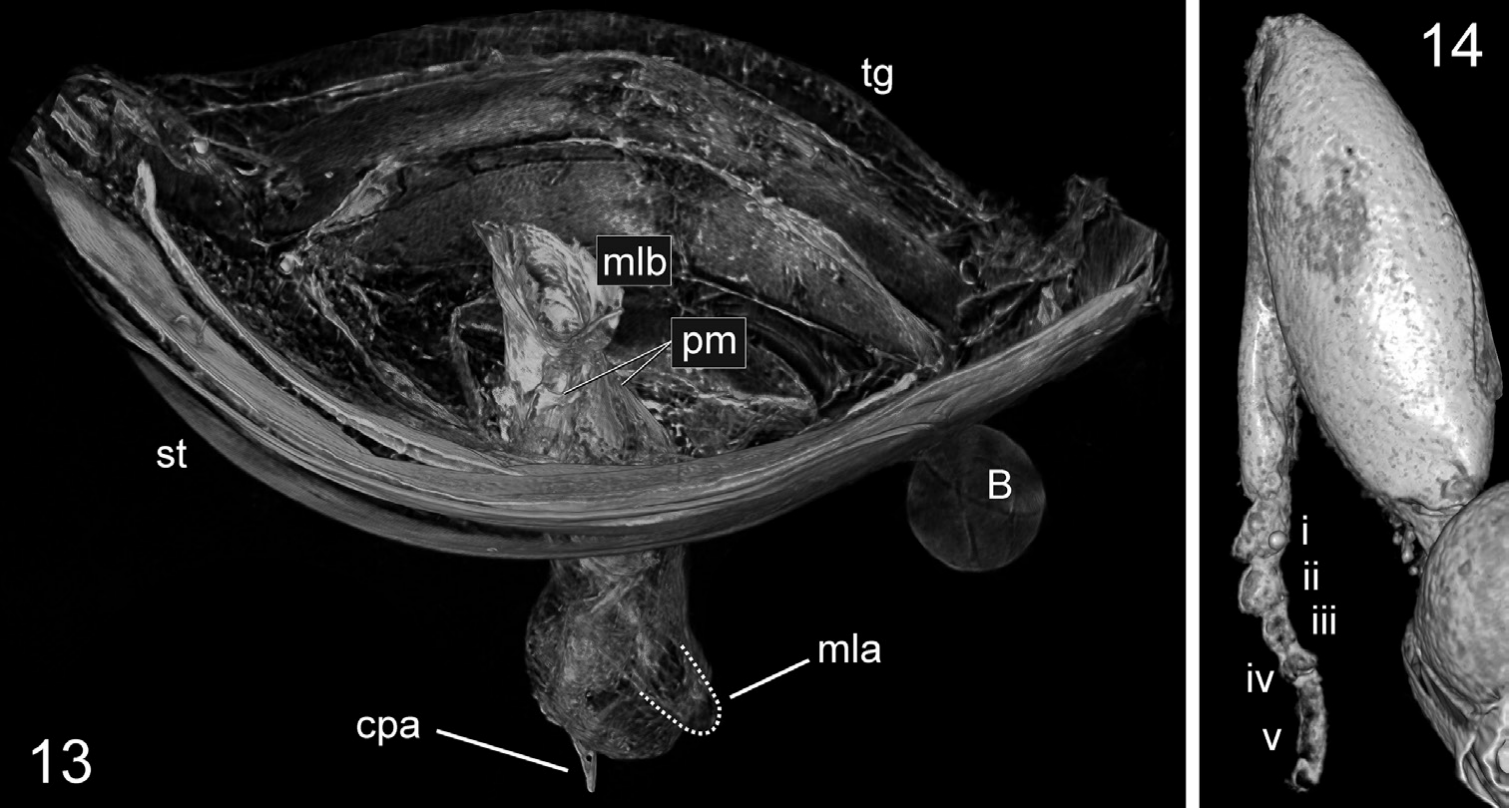
*Trechus exhibitorius* sp. n., holotype, volume rendering of selected body parts. **13** frontal view inside the abdomen showing the aedeagus which is fixed between the last abdominal segments **14** right front leg with protarsi i-v. Abbreviations: B = air bubble; cpa = apex of the longer copulatory piece; mla = median lobe apex; mlb = basal bulb of median lobe; pm = parameres; st = sternites; tg = tergites.

The amber piece is markedly darkened, and its surface shows several fine cracks, very probably as a result of its extended exposure to air. The beetle body is partly covered by milky coating and thus its right ventral surface and the mouth parts cannot be investigated by light microscopy (Figs 2, 3). The specimen is slightly shrunken with parts of its exoskeleton, which is thus dissociated from the inclusion wall. The latter represents the negative imprint of the beetle body and could be imaged together with the detached parts of the beetle exoskeleton using micro-CT, e.g., the detached margins of the pronotum (Fig. 4). Parts of the exoskeleton are broken along sutures and impressions, e.g., pronotal median longitudinal impression (Fig. 4), gula, occipital impression, mentum/submentum (if not naturally so), prosternum/proepisternum (Figs 5, 6, 7). The aedeagus is partly moved out off the abdomen and attached in this position by the last abdominal segment; the endophallus with the claw-like apex of the copulatory piece is partly inflated (Fig. 12). At its base, the median lobe is completely disrupted from the sclerites of segment IX (genital ring); latter is lost including parts of the median lobe basal bulb and the left paramere (Fig. 13).

#### Syninclusions.

One Acari specimen (phoretic mite of Parasitidae?) attached on the right elytron of *Trechus exhibitorius*, stellate hairs, and numerous dirt particles.

#### Description.

Body length: 4.4 mm.

***Colour*:** The whole body surface appears blackish brown, very shiny; variation in colouration of the different parts of the beetle body is not recognizable.

***Microsculpture*:** Surface of head and elytra, disc of pronotum with shallowly engraved slightly transverse meshes, clypeus with slightly engraved almost isodiametric meshes, base and laterobasal furrows of pronotum with moderately engraved almost isodiametric meshes (magnification ×100).

***Head*:** Moderately large and transverse; length 0.94 mm. Mandibles moderately slender, the right one tridentate, with all denticles combined and subequally distributed, but with the apical one much more protruding than the second (Figs 7–9). Labrum with apical margin markedly concave, with three pairs of setae. Clypeus with two pairs of setae in normal position. Shape of apical segments of maxillary and labial palpi as in *Trechus* sensu stricto. Mentum and submentum completely divided by a distinct suture (artefact? Fig. 7). Eyes (Fig. 6) rather small, flat; tempora about half as long as eyes, convex, moderately wrinkled to the neck, smooth. Frons and supraorbital area smooth, markedly convex, with supraorbital furrows deep, complete, uniformly bent on disc (Fig. 4); two supraorbital setae present and in normal position for *Trechus*. Antennae moderately slender, with three antennomeres extending beyond the pronotal base; scapus robust, 1.15 times longer and 1.3 times broader than pedicellus, 1.1 times longer than third antennomere; third and fourth antennomeres of the same length.

***Prothorax*:** Pronotum rather large, transverse (width/length = 1.47), length 0.84 mm, 1.53 times broader than head, broadest somewhat before middle, with sides evenly rounded in anterior 2/3 and straight before laterobasal angles; latter small, almost rectangular, not protruded laterally. Basal margin 1.22 times broader than apical margin. Disk markedly convex, smooth. Anterior margin straight in middle, lateroapical angles slightly protruded, rounded. Posterior margin not beaded, slightly convex in middle, markedly incised towards outer quarters, not shifted anteriorly at basolateral angles. Median longitudinal impression distinct, not deepened near base, disappearing near apex; anterior transverse impression very fine, smooth; posterior transverse impression linear, deep on sides, shallower in middle, smooth; laterobasal foveae indistinct, smooth. Lateral groove moderately narrow throughout, very slightly widened towards base, smooth. Both lateral and laterobasal setae present, with the lateral seta located near apical third of pronotum. Proepisternum glabrous, smooth.

***Pterothorax*:** Elytra markedly convex on disc, in dorsal view narrow ovate, length 2.58 mm, length/width = 1.50, widest near their mid-length, moderately wider than pronotum (width of elytra/width of pronotum = 1.30), glabrous beside normal setation. Humeral angles fully rounded, basal groove absent. Parascutellar stria moderately long, parascutellar seta present. Striae finely punctate apart from the apical fifth of elytra; first stria complete, outer striae disappearing near apex; three inner striae deeply impressed with intervals convex, fourth stria finer, fifth stria very fine, sixth and seventh striae absent, eighth stria finely impressed from level of the medial setae of the umbilical series and deeply impressed from level of the preapical group. Each elytron with two discal setae in third stria, and with preapical seta, located in the apical cross of the second and third striae, slightly closer to the suture than to the apical margin of elytra (Fig. 10). Recurrent stria rather short, extending towards the reduced fifth stria anteriorly, hardly bowed, with its front end situated at level of the preapical seta (Fig. 10). Setae of umbilicate humeral series close to the elytral margin, with four setae almost equidistant from each other; setae of the medial group of the umbilicate series far removed from the preapical group (Figs 6, 12). Metepisternum short, glabrous and smooth, with outer margin 1.3 times longer than anterior margin (Fig. 11).

***Abdomen*:** Abdominal sternites V–VII each with one (male) pair of setae near apical margin; surfaces smooth, without hairs or micropunctures.

***Legs*:** Moderately robust, all femora unmodified; protibiae straight and moderately dilated towards apex (structures on protibial surface are not visible using light microscopy due to milky coating, and could not be imaged using micro-CT). Basal two protarsomeres moderately dilated (Fig. 14).

**Figure 15.**
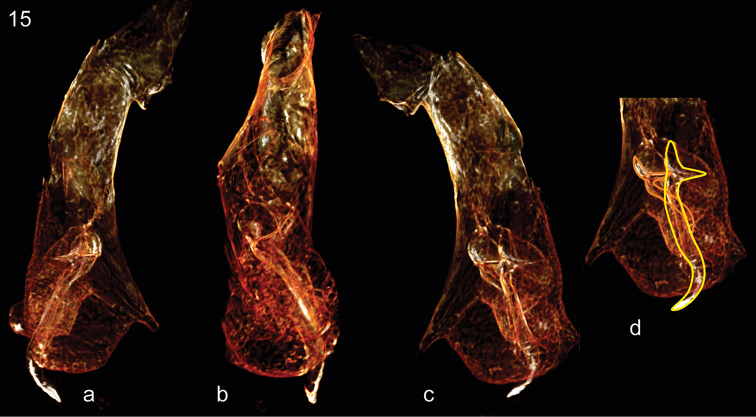
*Trechus exhibitorius* sp. n., holotype, volume rendering of the aedeagal median lobe with endophallus. **a** right lateral aspect **b** dorsal aspect **c** left lateral aspect **d**, same as **c**, but with contours of the two copulatory pieces indicated by coloured lines.

***Male genitalia*** (Figs 13, 15, Movie 1): Median lobe in apical half fully closed on dorsal side, in lateral view evenly bent throughout, with basal bulb of average size for *Trechus* (bulb is destroyed on its left side including left paramere missing); apical lamella moderately long, simple, conically tapering. Endophallus armed with two copulatory pieces, one is long rod-shaped and twisted, angularly curved and pointed apically, the other is less distinct, mainly due to its ambiguous distal portion, but with proximal portion semi-circular, very similar in shape and size to that of the first piece.

**Figure 16.**
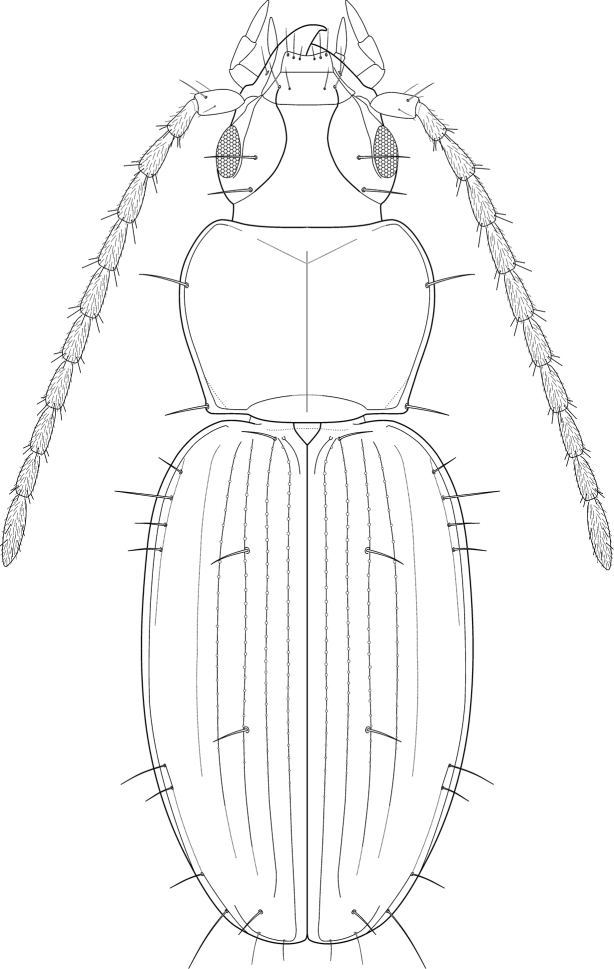
*Trechus exhibitorius* sp. n., reconstruction of the external shape in dorsal view.

**Movie 1.**
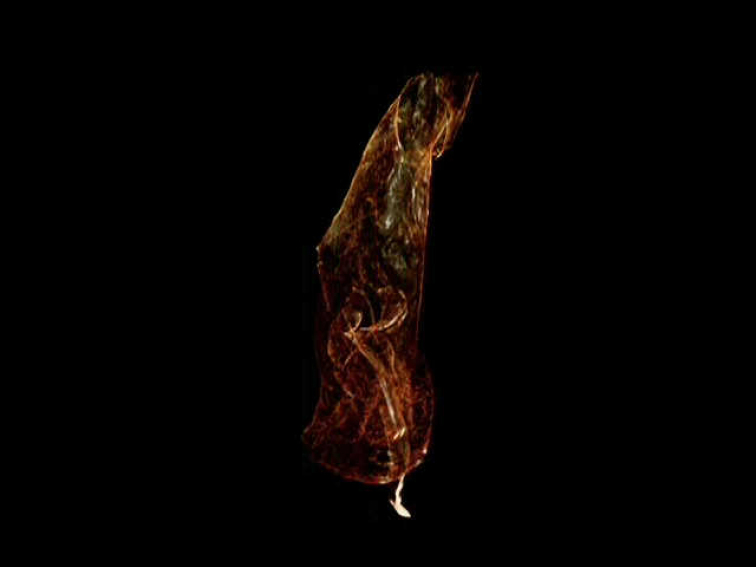
*Trechus exhibitorius* sp. n., holotype, volume rendering of the aedeagal median lobe with endophallus. YouTube link: https://youtu.be/u7hzlyrxOTA

#### Derivatio nominis.

Species epithet is derived from the Latin term “exhibitorius” = exhibitor. This name was given under the impression that the new species presents itself in a rather vulgar way.

#### Relationships.

Some characters of the pronotal structure, namely the missing laterobasal foveae coupled with rectilinear lateral portions of the basal transverse impression seem to be most useful for correct interpretation of the systematic position of the species in question. These two character states are usually correlated, although in some extant groups, the sublinear basal transverse impression may combine with the moderately impressed laterobasal foveae, the latter being separated from the lateral groove by clearly convex portions of the basal slope of the pronotal disc (e.g. *Trechus balkaricus* Belousov, 1990 and *Trechus fusculus* Motschulsky, 1850 with numerous related Caucasian and Turkish species). The boundary between the basal foveae completely missing and barely detectable is rather subjective and is often difficult to be recognized even in extant species. However, the combination of these two characters in the amber species makes a rather reliable basis for further considerations. In this respect, *Trechus exhibitorius* sp. n. resembles members of the *obtusiusculus* species group from the Balcans, southern Alps and Carpathians (some Caucasian species, such as *Trechus fischtensis* Reitter, 1888, seem to be also closely related to this group), the *montanellus* and *liopleurus* species group sensu lato (Belousov, 1987), the former from the Alps, Beskids and Sudetes, the latter from the Caucasus and Turkey. We deliberately neglected the two other groups with a similar structure of the pronotum: the *tingitanus* and *quadristriatus* (incuding *Trechus obtusus* Ericson, 1837 and relied taxa) species groups since the *tingitanus* group differs drastically by having deeper and strongly punctured exterior elytral striae while the *quadristriatus* group has a clearly convex pronotal basal margin with lateral portions oblique.

In the following, we will discuss our findings with the three remaining species groups. Members of the *obtusiusculus* species group and related Caucasian taxa, all differ readily from *Trechus exhibitorius* sp. n. in the endophallus armature consisting of a simple, anisotopous, scapus-like plate and therefore do not seem to be directly related to the species in question. The situation becomes much more interesting with regard to the *montanellus* and *liopleurus* groups. Jeannel (1927) considered both groups to be of Asian origin. This assumption seems to be confirmed with the description of *Trechus mordkovitschi* Shilenkov, 1982 and especially *Trechus shilenkovi* Belousov & Kabak, 1992, both from Siberia which are very similar to members of these two groups. According to Jeannel (1927) the differences between the *montanellus* and *liopleurus* groups mainly concern the degree of development of the external elytral striae and therefore, the conjunction of the apical recurrent stria with the fifth elytral stria which is clear in members of the *montanellus* species group and indistinct, with the apical striole abruptly interrupted anteriorly and external stria much more reduced, in members of the *liopleurus* species group. Though this observation is correct for many species of the groups under consideration, it is not present in all species. The Caucasian *liopleurus* species group is very heterogeneous and, doubtless, deserves to be split in a number of more natural species subgroups. Within each of these subgroups, we can see similar evolutionary patterns resulting in appearance of large-sized wingless species with parallel-sided habitus, shallow external elytral striae, narrow and parallel internal interspaces and massive pronotum characterized by the basal transverse impression subrectilinear and sharply engraved as well as the basolateral foveae nearly missing. On the other hand, some members of the *liopleurus* group have an ovate habitus, similar to that of members of the *montanellus* group, external elytral striae well-impressed, with clear conjunction of stria 5 with the apical recurrent stria and the pronotum with distinct basolateral foveae and the basal transverse impression which is, though sharply engraved, clearly bent laterally. Among these species, *Trechus gagrensis* Jeannel, 1927 is especially of high interest, since it is, doubtless, most closely related to *Trechus liopleurus* Chaudoir, 1850. However, the pronotal structure similar to that of *Trechus exhibitorius* sp. n. is observed also in some species related to *Trechus balkaricus* within the *maculicornis* species group as defined by Jeannel (1960) and some species of the *fusculus* (= *bradycelloides*) group (Jeannel 1960; Pawłowski 1979), despite the fact that most of species assigned to these groups have the pronotum with distinct laterobasal foveae and the basal transverse impression rather shallow and clearly bent. To summarize, we can infer that the pronotal characters listed above might have evolved independently and might be associated with a similar mode of life; their phylogenetic importance should be cautiously considered.

Fortunately, the fossil in question is the first extinct *Trechus* species with the aedeagus and endophallus armature well preserved. As to the aedeagus shape, its apical portion, slightly attenuated downwards, is very similar to that of both known species of the *montanellus* species group, *Trechus shilenkovi* from the Altai mountains and members of the *alpigradus* subgroup within the *liopleurus* group, especially *Trechus arnoldii* Belousov, 1987 from the West Caucasus. The observed structure of the endophallus armature is challenging to interpret, especially bearing in mind that it was fixed half-way inflated. However, we can clearly see one piece which is long and twisted, angularly curved and pointed apically. A large, branch-like and twisted copulatory piece is rather common in the endophallus armature of many *Trechus* species groups. In addition to the *Trechus sylvicola* K. Daniel & J. Daniel, 1898 from the *montanellus* group listed above, some Turkish species of the *fusculus* group are worth to be mentioned, for example: *Trechus michaeli* Pawlowski 1978 and *Trechus ziganensis* Jeanne, 1976. Though both of these species have the pronotum with rather distinct laterobasal foveae and less sharp transverse basal impression, some related species have the pronotum structure quite similar to that of *Trechus exhibitorius* sp. n. The second piece of the endophallus armature of this species is less distinct, mainly due to its ambiguous distal portion. However, its proximal portion is clearly detectable, semi-circular, and very similar in shape and size to that of the first piece (Fig. 15d). This fact deserves careful consideration. Examination of the inflated endophallus in *Trechus* species shows that in some *Trechus* lineages, including those addressed by this paper, the most primitive structure of the endophallus armature consists of two branch-like similar pieces attached to the proximal end of the endophallus (becoming the distal end when inflated) (unpublished data I.B.). Their basal portions seem to be semi-circular in projection, but are in fact semi-spherical to ensure their firm position on the convex surface of the inflated endophallus (such a condition is typical of members of the *maculicornis* species group). Their evolution resembles a development of the right piece coupled with progressive reduction of the left one. In this case, the basal portion of the left piece becomes either much smaller or is incorporated (at least, topologically) in the basal portion of the right piece. This evolution of the endophallus armature seemed to take place independently in various *Trechus* lineages. If the above interpretation of the endophallus armature in *Trechus exhibitorius* sp. n. is correct, this species combines a rather primitive structure of the endophallus armature and aedeagus (including an average size of the aedeagus vs. hypertrophied in strongly evolved species) with some specialized external characters commonly found in mesophylous *Trechus*. It should likely be placed near the basal portion of the phylogenetic stem branching into the *montanellus*, *liopleurus*, *fusculus* and, probably, *osmanilis* and *maculicornis* species groups. However, monophyly of the above mentioned species groups needs to be tested using molecular methods.

#### Differential diagnosis.

Two additional *Trechus* fossils were described from the Eocene Baltic amber: *Trechus balticus* and *Trechus eoanophthalmus* (Schmidt and Faille 2015, Schmidt et al. 2016). *Trechus exhibitorius* sp. n. is easily distinguished from the blind *Trechus eoanophthalmus* by the fully developed eyes and the much slender body. *Trechus balticus* is distinctly smaller (3.6 mm versus 4.4 mm in *Trechus exhibitorius* sp. n.), has a stouter body with broader head and shorter elytra, larger and deeper impressed micro-meshes on disc of head and more markedly reduced external elytral striae.

## Conclusions

Among the few Carabidae species described from Baltic amber on species level three species are now attributed to the genus *Trechus*. All these species are obligatory wingless characterized by markedly shortened metepisternae and fully rounded humeri, and it can therefore be presumed that these Eocene species lived on the forest floor along mountain slopes, just as it is known for the huge number of wingless trechine beetles today (Schmidt et al. 2016). This conclusion is in contradiction with the assumption of Alekseev and Alekseev (2016) that the amber forest was formed on a plain or slightly hilly area. Moreover, considering the low dispersal ability of the tiny wingless beetles within their mountainous environment, very small distributional areas can be expected for each of the species and therefore the contemporary existence of a large number of closely related allopatric *Trechus* species (Schmidt et al. 2016). This scenario makes the discovery of additional wingless *Trechus* species preserved in Baltic amber highly likely. Thus, trechine beetles gain interest especially with regard to phylogeographic studies including dating of the phylogeny of the respective group (e.g., Faille et al. 2013, 2014, 2015). In this respect the discovery of *Trechus exhibitorius* sp. n. is a stroke of luck since fossil external and internal characters of the male genitalia could be investigated for the first time and thus, the systematic position of this species within the megadiverse *Trechus* sensu lato can be more certainly assigned than it has been possible for the previously described fossils. In this respect one of the most relevant conclusions based on the finding of *Trechus exhibitorius* sp. n. is that the *Trechus* clade as verified by Faille et al. (2013) seems at least as old as the Eocene with a minimum age of approx. 35–50 Mya (Standke 2008). Therefore, a distinctly faster molecular clock in *Trechus* compared to other arthropods is highly improbable, as it was estimated by Contreras-Diaz (2007) in a study of *Trechus* endemic to the Canary Islands and subsequently used by Lhose et al. (2011) in an analysis of the quaternary distributional history of *Trechus* from the Alps.

## Supplementary Material

XML Treatment for Trechus exhibitorius
